# Cinchonidine, a Natural Quinoline Alkaloid, Attenuates Ischemic Neurovascular Injury Through Blood–Brain Barrier Preservation

**DOI:** 10.3390/biomedicines14071442

**Published:** 2026-06-25

**Authors:** Kuan-Jung Lu, Chia-Yuan Hsu, Thanasekaran Jayakumar, Cheng-Ying Hsieh, Ruei-Dun Teng

**Affiliations:** 1 Department of Pharmacology, School of Medicine, College of Medicine, Taipei Medical University, Taipei 11031, Taiwan; lucindalu19950107@gmail.com; 2 Graduate Institute of Medical Sciences, College of Medicine, Taipei Medical University, Taipei 11031, Taiwan; gordanmike1003@gmail.com; 3 Department of Ecology and Environmental Sciences, Pondicherry University, Puducherry 605014, India; tjayakumar@pondiuni.ac.in; 4 Department of Medical Research, Taipei Medical University Hospital, Taipei 110031, Taiwan

**Keywords:** natural bioactive compounds, natural products, translational research, ischemic brain injury, blood–brain barrier, NVU, cinchonidine

## Abstract

**Background/Objectives**: Ischemic stroke remains a major global health challenge, yet therapeutic options are severely restricted by narrow treatment windows and the risk of hemorrhagic transformation. Natural small molecules represent a valuable reservoir for discovering novel neuroprotective leads with favorable safety profiles. Cinchonidine, a natural quinoline alkaloid, has shown anti-inflammatory and cytoprotective properties, but its potential in treating ischemic stroke is largely unexplored. This study aimed to evaluate the neurovascular protective effects and hemostatic safety of cinchonidine in preclinical stroke models. **Methods**: We evaluated cinchonidine using a mouse model of middle cerebral artery occlusion (MCAO) and in vitro oxygen–glucose deprivation (OGD) models in cerebral endothelial cells (CECs) and Neuro2A cells. Infarct volume, brain edema, and neurological recovery were assessed. Blood–brain barrier (BBB) integrity was measured via Evans blue extravasation. Mechanistic markers, including microglial activation, pro-inflammatory mediators (iNOS, COX-2), and apoptosis-related signaling, were examined. Additionally, cinchonidine’s effect on platelet aggregation was also tested. **Results**: Cinchonidine significantly reduced infarct volume and brain edema while improving neurological functional recovery. It effectively preserved BBB integrity and enhanced cell viability under OGD conditions. Furthermore, cinchonidine suppressed microglial activation and decreased the expression of pro-inflammatory mediators. These protective effects were associated with the modulation of apoptotic signaling pathways. These protective effects were accompanied by reduced p53-associated stress signaling in endothelial cells and ischemic brain tissue. Importantly, cinchonidine did not significantly interfere with platelet aggregation, suggesting a potentially favorable hemostatic profile. **Conclusions**: Cinchonidine attenuates ischemic brain injury and is associated with endothelial protection, preservation of BBB integrity, and modulation of inflammatory and apoptotic responses. As a natural lead compound that does not compromise hemostasis, cinchonidine represents a promising lead compound for further development as a neurovascular protective strategy in ischemic stroke.

## 1. Introduction

Stroke, also known as cerebrovascular accident (CVA), is a major neurological disorder characterized by the sudden loss of blood supply to brain tissue, resulting in localized ischemia and subsequent neuronal injury [1]. It remains one of the leading causes of mortality and long-term disability worldwide, posing a substantial global health burden [2]. Epidemiological studies estimate that approximately six million individuals die from stroke each year, and a significant proportion of survivors experience persistent neurological deficits, including motor dysfunction, cognitive impairment, and reduced quality of life [3,4]. As the global population continues to age, the incidence and prevalence of stroke are expected to increase, further amplifying its societal and economic impact. Therefore, the development of effective therapeutic strategies for ischemic stroke remains an urgent and unmet medical need [5]. Currently, recombinant tissue plasminogen activator (rt-PA) is the only widely approved pharmacological treatment for acute ischemic stroke [6]. rt-PA functions by dissolving thrombi and restoring cerebral blood flow; however, its clinical application is severely limited by a narrow therapeutic window of 3–4.5 h following stroke onset. Administration beyond this time frame significantly increases the risk of hemorrhagic transformation, thereby restricting its use to a small subset of patients [7]. In addition, reperfusion following ischemia can paradoxically exacerbate tissue damage through mechanisms collectively referred to as ischemia–reperfusion injury. These processes involve oxidative stress, inflammation, and apoptotic signaling [8], which contribute to further neuronal and vascular damage [9]. Thus, there is a critical need for therapeutic agents that not only restore blood flow but also protect brain tissue from secondary injury. Among the various pathological processes involved in ischemic stroke, disruption of the blood–brain barrier (BBB) has emerged as a key determinant of disease progression and outcome [10]. The BBB is a highly specialized structure composed primarily of endothelial cells, pericytes, and astrocytic end-feet, which collectively regulate the exchange of molecules between the bloodstream and the central nervous system [11]. Under physiological conditions, the BBB maintains brain homeostasis by tightly controlling vascular permeability [12]. However, during ischemic injury, endothelial dysfunction leads to increased permeability, resulting in vasogenic edema, infiltration of inflammatory cells, and exacerbation of neuronal damage. Therefore, preservation of endothelial integrity and BBB function represents a promising therapeutic target for mitigating ischemic brain injury. Endothelial cells, as the structural and functional core of the BBB, are particularly vulnerable to ischemic stress [13]. Oxygen–glucose deprivation triggers a cascade of cellular events, including oxidative stress, mitochondrial dysfunction, and activation of apoptotic pathways, ultimately leading to endothelial cell death and barrier breakdown [14]. In addition, endothelial dysfunction promotes the release of pro-inflammatory mediators, further amplifying neuroinflammation and tissue injury. Given this central role, strategies aimed at protecting endothelial cells may provide dual benefits by maintaining vascular integrity and reducing inflammatory responses. However, current stroke therapies have largely focused on thrombolysis and neuroprotection, with relatively limited attention to endothelial-targeted interventions. Cinchonidine, a natural alkaloid derived from the bark of *Cinchona* species, has attracted increasing attention due to its diverse pharmacological properties. Along with other cinchona alkaloids such as quinine and quinidine, cinchonidine has historically been used in the treatment of malaria and certain cardiovascular conditions. Although cinchonidine, quinine, and quinidine share a common quinoline backbone, they differ in stereochemical configuration and pharmacological properties. Quinine is primarily recognized for its antimalarial activity, whereas quinidine has been widely used as a class IA antiarrhythmic agent [15]. In contrast, the biological activities of cinchonidine have been comparatively less explored, particularly in ischemic stroke, despite increasing evidence supporting its anti-inflammatory and cytoprotective effects. Natural alkaloids have emerged as promising candidates for ischemic stroke therapy because of their anti-inflammatory, antioxidant, and anti-apoptotic activities. However, most studies have focused on compounds such as berberine, matrine, and tetrandrine, while the neurovascular protective potential of cinchonidine remains largely unexplored. Recent studies have suggested that cinchonidine possesses anti-inflammatory, antioxidant, and cytoprotective effects. Notably, emerging evidence indicates that cinchonidine may protect endothelial cells from injury by modulating intracellular signaling pathways, including those involving inflammatory and apoptosis pathway [16]. The p53-mediated apoptotic response is a documented feature of ischemic endothelial dysfunction. Understanding how cinchonidine modulates such cellular stress markers, alongside its evaluation in hemostatic safety, is crucial for determining its viability as a multi-functional therapeutic candidate for stroke [17]. Among the cinchona alkaloids, cinchonidine stands out with the potential to mitigate ischemia-induced endothelial injury compared to other quinine-related compounds [18]. In the present study, we investigated the therapeutic potential of cinchonidine, a natural quinoline alkaloid, using a mouse model of middle cerebral artery occlusion (MCAO) and in vitro models of ischemic injury. We hypothesized that cinchonidine exerts robust neurovascular protective effects by preserving endothelial function and maintaining BBB integrity. To test this hypothesis, we evaluated infarct size, neurological recovery, and neuroinflammatory responses, while exploring its impact on endothelial cell survival and associated apoptotic signaling pathways. Furthermore, given the clinical limitations of current thrombolytic therapies, we specifically assessed the effects of cinchonidine on platelet aggregation examine its hemostasis profile. Our findings provide new insights into the vascular protective mechanisms of cinchonidine and highlight its potential as a promising and safe natural lead for the treatment of ischemic stroke.

## 2. Materials and Methods

### 2.1. Ethics Statement

All animal experiments were approved by the Taipei Medical University Institutional Animal Care and Use Committee (IACUC; Approval No. LAC2022-0542, approved on 13 April 2023) and were conducted in accordance with institutional guidelines and ARRIVE guidelines.

### 2.2. Experimental Animals Randomized and Blinding

Male C57BL/6 mice (6–8 weeks old) were obtained from BioLASCO Taiwan Co., Ltd. (Taipei, Taiwan). Animals were housed under controlled environmental conditions (23 ± 1 °C) with a 12 h light/dark cycle (lights on at 07:00) and were provided ad libitum access to standard laboratory chow and water. All experimental procedures were performed between 08:00 and 12:00 to minimize circadian variation. Male C57BL/6 mice (6–8 weeks old) were randomly assigned to experimental groups using a computer-generated randomization sequence. Investigators responsible for behavioral assessments, infarct volume quantification, immunofluorescence analysis, and Western blot quantification were blinded to treatment allocation throughout data acquisition and analysis. Separate cohorts of animals were used for acute histological analyses and behavioral assessments. For the acute injury cohort, mice were euthanized 24 h after reperfusion for TTC staining, brain edema measurement, Evans blue extravasation, immunofluorescence staining, qPCR analysis, and biochemical studies. Because TTC analysis requires tissue collection, these animals were not included in behavioral evaluations. A separate behavioral cohort was used for neurological assessments, including the Neurological Severity Score (NSS), rotarod performance, and locomotor activity testing conducted before MCAO and at 24 and 48 h after reperfusion. For both cohorts, animals were randomly assigned to the following groups: Sham, Vehicle, Cinchonidine pre-treatment (5 mg/kg), Cinchonidine pre-treatment (10 mg/kg), and Cinchonidine post-treatment (10 mg/kg). Animals exhibiting surgical complications, signs of infection, or unsuccessful induction of ischemia were excluded according to predefined exclusion criteria established before study initiation.

### 2.3. Middle Cerebral Artery Occlusion (MCAO) Model

Male C57BL/6 mice (6–8 week old) were anesthetized with 3% isoflurane in a gas mixture of 75% air and 25% oxygen. Body temperature was maintained at 37.5 ± 0.5 °C using a temperature-controlled heating pad. Transient focal cerebral ischemia was induced using the middle cerebral artery occlusion (MCAO) model as previously described [19]. Briefly, the right common carotid artery was exposed, and Doccol Corporation, Sharon, MA, USA; diameter (0.21–0.22 mm) coated with a 3 mm silicone-coated tip was inserted from the external carotid artery into the internal carotid artery until it occluded the origin of the middle cerebral artery. After 30 min of occlusion, the filament was carefully withdrawn under brief anesthesia to allow reperfusion. The surgical incision was then closed, and the mice were allowed to recover from anesthesia.

### 2.4. Cinchonidine Administration

Cinchonidine (96% purity; Sigma-Aldrich, St. Louis, MO, USA; Cat. No. C80407) was dissolved in a vehicle consisting of Polyethylene glycol 400 (PEG400, S Sigma-Aldrich, St. Louis, MO, USA) and DMSO (9:1, *v*/*v*). The vehicle control group received the same formulation without cinchonidine. Cinchonidine was administered intraperitoneally (i.p.) at a volume of 100 μL per mouse. The doses of 5 and 10 mg/kg were selected based on preliminary dose-finding studies and previous reports describing the biological activities of quinoline alkaloids. For pre-treatment experiments, cinchonidine was administered 1 h before MCAO induction. For post-treatment experiments, cinchonidine (10 mg/kg) was administered 1 h after reperfusion.

### 2.5. Neurological Severity Score (NSS) Assessment

Neurological function was evaluated using the neurological severity score (NSS) system. Assessments were performed prior to MCAO and at 24 and 48 h post-injury. The NSS is based on an 18-point scale (0 = normal; 18 = maximal deficit) and includes evaluations of motor function, sensory function, reflexes, and balance. A score of 1 is assigned for failure to perform a task or absence of a tested reflex. Therefore, higher NSS values indicate more severe neurological impairment.

### 2.6. Spontaneous Locomotor Activity and Rotarod Test

Prior to behavioral testing, mice were trained on an accelerating rotarod for 3 consecutive days. The rotarod speed increased from 4 to 40 rpm over 5 min, with increments of 4 rpm every 30 s. Spontaneous locomotor activity (LMA) was assessed before MCAO and at 24 and 48 h after MCAO using an automated activity monitoring system (Noldus Information Technology, Wageningen, The Netherlands). Locomotor activity was quantified by counting beam breaks over a 15 min period. Following LMA assessment, motor coordination and balance were evaluated using a rotarod apparatus (UGO Basile, Varese, Italy). The rotarod accelerated from 4 to 40 rpm over 3 min. The latency to fall was recorded for each mouse, with a maximum cutoff time of 180 s.

### 2.7. Immunofluorescence Staining

Brain sections were prepared and stained as previously described [20]. Briefly, sections were permeabilized with 0.2% Triton X-100 in phosphate-buffered saline (PBS) and then incubated with primary antibodies diluted in PBS containing 5% normal goat serum for 18–24 h at 4 °C. Images were acquired using an EVOS FL Cell Imaging System (Thermo Fisher Scientific, Waltham, MA, USA). To identify specific cell types, the following primary antibodies were used: ionized calcium-binding adaptor molecule 1 (Iba-1; Wako Pure Chemical Industries, Osaka, Japan) for microglia. Activated microglia were identified based on enlarged cell bodies and reduced process complexity. The percentage of activated Iba1-positive cells was quantified from three randomly selected fields per section using ImageJ software 1.54g. CD31 (R&D SYSTEMS, Minneapolis, MN, USA), Claudin-5 (Invitrogen, Carlsbad, CA, USA), p53 expression was detected using an anti-p53 antibody (Abcam, Cambridge, UK).

### 2.8. TTC Staining and Measurement of Infarct Volume and Edema

Mice were deeply anesthetized and transcardially perfused with 50 mL of ice-cold phosphate-buffered saline (PBS). Brains were then rapidly removed and rinsed in cold PBS. The brains were sliced into 1 mm thick coronal sections using a brain matrix. Six sections spanning from the olfactory bulb to the cerebellum were collected and incubated in 2% 2,3,5-triphenyltetrazolium chloride (TTC; Sigma-Aldrich, St. Louis, MO, USA) at 37 °C for 15–20 min in the dark. After staining, sections were fixed in 4% formalin for 1 h. Stained brain sections were imaged using a digital camera. Infarct area and brain edema were analyzed in a blinded manner using ImageJ software (NIH, Bethesda, MD, USA). Infarct volume was calculated by integrating infarct areas across all sections. To correct for brain edema, infarct volume was expressed as a percentage of the contralateral hemisphere using the following formula: Corrected infarct volume (%) = [(contralateral hemisphere area—ipsilateral non-infarcted area)/contralateral hemisphere area] × 100 [21].

### 2.9. BV2 Cell Culture and LPS Stimulation

BV2 microglial cells were cultured in Dulbecco’s Modified Eagle Medium (DMEM) (ThermoFisher, Waltham, MA, USA) supplemented with 10% fetal bovine serum (FBS) and 1% penicillin–streptomycin at 37 °C in a humidified incubator containing 5% CO_2_. For inflammatory stimulation, BV2 cells were seeded at a density of 5 × 10^5^ cells/well in 6-well plates and pretreated with cinchonidine (1, 5, or 10 μM) or vehicle for 1 h prior to lipopolysaccharide (LPS; 150 ng/mL) stimulation. Cells were subsequently incubated with LPS for 24 h.

### 2.10. Western Blotting Assay

Protein samples were extracted from tissue and mixed with sodium dodecyl sulfate (SDS) loading buffer. Equal amounts of protein (35 µg) were separated by SDS–polyacrylamide gel electrophoresis (SDS-PAGE) and subsequently transferred onto polyvinylidene difluoride (PVDF) membranes. Membranes were blocked with 5% skim milk in TBST buffer (10 mM Tris-base, 100 mM NaCl, and 0.01% Tween 20) for 1 h at room temperature and then washed three times with TBST. The membranes were incubated with primary antibodies: Cleaved-PARP (Cell Signaling Technology, Danvers, MA, USA), COX-2 (R&D SYSTEMS, Minneapolis, MN, USA), iNOS (Cell Signaling Technology, Danvers, MA, USA) and α-Tubulin (Sigma-Aldrich, Burlington, MA, USA) overnight at 4 °C, followed by incubation with appropriate secondary antibodies for 1 h at room temperature. Protein bands were visualized using an enhanced chemiluminescence (ECL) detection system. Band intensities were quantified using ImageJ software (NIH, Bethesda, MD, USA).

### 2.11. Quantitative Real-Time PCR (RT-qPCR)

Total RNA was isolated using the RNeasy Mini Kit (QIAGEN, Hilden, Germany) according to the manufacturer’s instructions. RNA concentration and purity were determined using spectrophotometric analysis. For cDNA synthesis, 1 μg of total RNA was reverse-transcribed using the SuperScript™ IV First-Strand Synthesis System (Invitrogen, Carlsbad, CA, USA). Quantitative real-time PCR (RT-qPCR) was performed using Fast SYBR™ Green Master Mix (Thermo Fisher Scientific, Waltham, MA, USA) on a StepOne™ Real-Time PCR System (Applied Biosystems, Foster City, CA, USA). The amplification protocol consisted of an initial denaturation step at 95 °C for 20 s, followed by 45 cycles of denaturation at 95 °C for 5 s and annealing/extension at 60 °C for 30 s. Relative gene expression levels were calculated using the 2^−ΔΔCt^ method. GAPDH was used as the endogenous reference gene, and all target genes were normalized to GAPDH expression. Primer sequences used for RT-qPCR are listed in Table 1.

### 2.12. Human Platelet Preparation

This study was conducted in accordance with the ethical principles of the Declaration of Helsinki and was approved by the Institutional Review Board of Taipei Medical University (TMU-JIRB-N201812024, approved on 24 December 2024). Written informed consent was obtained from all participants prior to inclusion. Peripheral venous blood samples were collected from 30 healthy adult volunteers (male and female, aged 20–35 years). To minimize potential confounding effects on platelet function, individuals who had used antiplatelet agents, nonsteroidal anti-inflammatory drugs, dietary supplements, or other medications known to influence platelet activity within 14 days prior to blood collection were excluded. Washed human platelets (3.6 × 10^8^ cells/mL) were prepared from blood samples collected from 30 healthy volunteers, as previously described [22]. Briefly, whole blood was mixed with acid/citrate/glucose solution (9:1, *v*/*v*) and centrifuged to obtain platelet-rich plasma (PRP). The PRP was incubated with EDTA (2 mM) and heparin (6.4 U/mL) for 5 min, followed by centrifugation at 500× *g* for 10 min. The platelet pellet was resuspended in Tyrode’s solution and incubated at 37 °C for 10 min. After centrifugation, the washing procedure was repeated, and platelets were finally resuspended in Tyrode’s solution containing 3.5 mg/mL bovine serum albumin (BSA). Platelet counts were determined using a Coulter counter (Beckman Coulter, Miami, FL, USA). The final Ca^2+^ concentration in the Tyrode’s solution was adjusted to 1 mM.

### 2.13. Platelet Aggregation Assay

Platelet aggregation was assessed using a Lumi-Aggregometer (Payton Associates, Scarborough, ON, Canada) following the protocol described by Chen et al. [22]. Platelet suspensions (3 × 10^8^ cells) were preincubated with various concentrations of cinchonidine and quinine (10–20µM) or phosphate-buffered saline (PBS; vehicle control) for 3 min prior to stimulation. Aggregation was then induced by the addition of collagen (2 µg/mL), and the reaction was monitored for 6 min. The extent of platelet aggregation was quantified as changes in light transmission.

### 2.14. Primary Mouse Cerebral Endothelial Cell Culture

Primary mouse cerebral endothelial cells (CECs) were cultured as previously described [21]. Cells were maintained in endothelial growth medium supplemented with growth factors at 37 °C in a humidified incubator containing 5% CO_2_.

### 2.15. Neuro2A Cell Culture

Neuro2A cells were cultured in DMEM supplemented with 10% fetal bovine serum and 1% penicillin–streptomycin at 37 °C in a humidified atmosphere containing 5% CO_2_.

### 2.16. Oxygen-Glucose Deprivation (OGD)

Confluent primary mouse cerebral endothelial cells (CECs) and Neuro2A cells were subjected to oxygen–glucose deprivation (OGD) as previously described, with minor modifications [23]. Briefly, culture medium was replaced with deoxygenated glucose-free HBSS, and cells were transferred to an anaerobic chamber containing 5% CO_2_, 10% H_2_, 85% N_2_, and 0.02–0.2% O_2_ for 4 h. Control cells were maintained under normoxic conditions.

### 2.17. Cell Viability, Cytotoxicity, and Apoptosis Assay

Neuro2a cells (1 × 10^4^ cells/well) and primary CECs (1 × 10^4^ cells/well) were seeded in 96-well white flat-bottom plates and incubated overnight at 37 °C. Cells were then went under oxygen–glucose deprivation (OGD) condition for 4.5 h. Cell viability, cytotoxicity, and apoptosis were evaluated using the ApoTox-Glo™ Triplex Assay (Promega, Madison, WI, USA) according to the manufacturer’s instructions. Fluorescence and luminescence signals were measured using a Varioskan Flash multimode microplate reader (Thermo Fisher Scientific, Waltham, MA, USA).

### 2.18. Statistical Analysis

All behavioral scoring, infarct quantification, immunofluorescence analyses, and molecular measurements were performed by investigators blinded to treatment allocation. Group identities were revealed only after completion of data analysis. All data are presented as mean ± standard error of the mean (SEM). Statistical analyses were performed using GraphPad Prism (version 9.02; GraphPad Software, San Diego, CA, USA). Comparisons among multiple groups were conducted using one-way analysis of variance (ANOVA) followed by Tukey’s post hoc test. A *p* value < 0.05 was considered statistically significant. Pearson correlation analysis was performed to evaluate correlation coefficients where appropriate.

## 3. Results

### 3.1. Cinchonidine Attenuates Cerebral Infarction and Brain Edema in a Mouse Model of Ischemic Stroke

**Figure 1:**
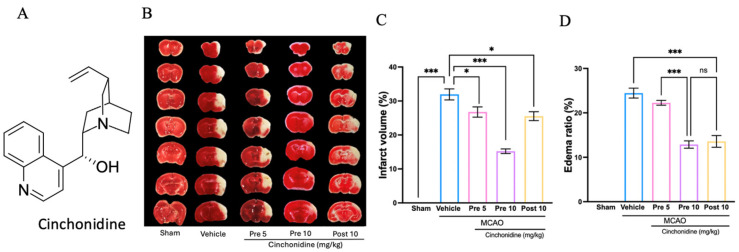
Cinchonidine attenuates cerebral infarction and brain edema in a mouse model of ischemic stroke. Mice were subjected to middle cerebral artery occlusion (MCAO) and treated with cinchonidine either 1 h before ischemia induction (Pre-5 and Pre-10 groups) or 1 h after reperfusion (Post-10 group). Brain tissues were harvested 24 h after reperfusion to evaluate acute ischemic injury. (**A**) Chemical structure of cinchonidine (C_19_H_22_N_2_O). (**B**) Representative images of 2,3,5-triphenyltetrazolium chloride (TTC)-stained coronal brain sections (1 mm thickness). White areas indicate infarcted tissue, whereas red areas indicate viable tissue. (**C**) Quantification of infarct volume, expressed as a percentage of the contralateral hemisphere after edema correction. (**D**) Quantification of brain edema, calculated as the volume difference between the ipsilateral and contralateral hemispheres. Data are expressed as mean ± SEM (*n* = 8 per group). * *p* < 0.05, *** *p* < 0.001 vs. vehicle-treated MCAO group, ns indicates no significant.

To evaluate both the prophylactic and therapeutic potential of cinchonidine, mice were treated with cinchonidine either 1 h before MCAO induction (Pre-5 and Pre-10 groups) or 1 h after reperfusion (Post-10 group). Representative TTC-stained brain sections showed a marked reduction in infarct size in cinchonidine-treated mice compared with the vehicle-treated MCAO group (Figure 1B). Quantitative analysis demonstrated that both pre-treatment and post-treatment with cinchonidine significantly reduced infarct volume (Figure 1C). Similarly, cinchonidine administration significantly attenuated brain edema, as indicated by a reduced edema ratio (Figure 1D). Notably, the neuroprotective effects observed in the post-treatment group suggest that cinchonidine retains efficacy even when administered after ischemic injury. These findings indicate that cinchonidine provides both preventive and therapeutic neuroprotection against ischemic brain injury.

### 3.2. Cinchonidine Promotes Functional Recovery and Improves Neurological Outcomes Following Ischemic Injury

**Figure 2:**
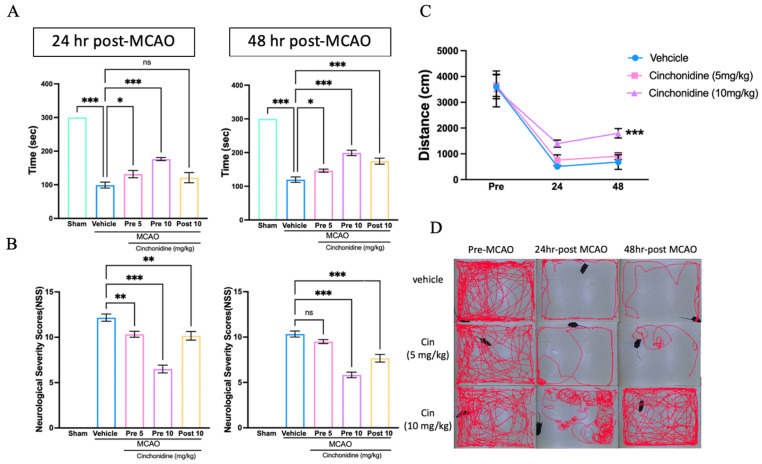
Cinchonidine promotes functional recovery and improves neurological outcomes following ischemic injury. Neurological function was assessed before MCAO and at 24 and 48 h after reperfusion to evaluate the effects of cinchonidine administered either 1 h before ischemia induction (Pre-5 and Pre-10 groups) or 1 h after reperfusion (Post-10 group). (**A**) Motor coordination and balance were evaluated using the rotarod performance test. The latency to fall was recorded and averaged from three trials. (**B**) Neurological deficits were assessed using the Neurological Severity Score (NSS), including motor, sensory, reflex, and balance functions. (**C**) Spontaneous locomotor activity was quantified as the total distance traveled in an open-field arena during a 15 min observation period. (**D**) Representative locomotor activity traces illustrating movement patterns before MCAO and at 24 and 48 h after reperfusion. Data are presented as mean ± SEM (*n* = 8 per group). * *p* < 0.05, ** *p* < 0.01, *** *p* < 0.001, as indicated, ns indicates no significant.

Neurological function was evaluated using rotarod performance, Neurological Severity Score (NSS), and spontaneous locomotor activity at 24 and 48 h after reperfusion. As shown in Figure 2A, MCAO markedly impaired motor coordination and balance, whereas cinchonidine treatment significantly improved rotarod performance. The Pre-10 group exhibited the greatest improvement at both 24 and 48 h after MCAO. Notably, mice receiving cinchonidine after reperfusion (Post-10 group) also showed improved motor performance compared with vehicle-treated MCAO mice, particularly at 48 h after injury. Consistent with these findings, NSS analysis demonstrated significantly reduced neurological deficits in cinchonidine-treated mice (Figure 2B). The Pre-10 group showed the lowest neurological deficit scores, while the Post-10 group also exhibited significant functional improvement relative to vehicle-treated animals at both time points. These findings indicate that cinchonidine improves neurological recovery when administered either before ischemia or after reperfusion. Furthermore, spontaneous locomotor activity was significantly increased following cinchonidine treatment, with the greatest recovery observed in the 10 mg/kg treatment group (Figure 2C). Representative locomotor activity traces are shown in Figure 2D. Collectively, these findings suggest that cinchonidine is associated with improved neurological recovery following ischemic stroke.

### 3.3. Cinchonidine Suppresses Neuroinflammatory Responses Without Affecting Platelet Aggregation

**Figure 3:**
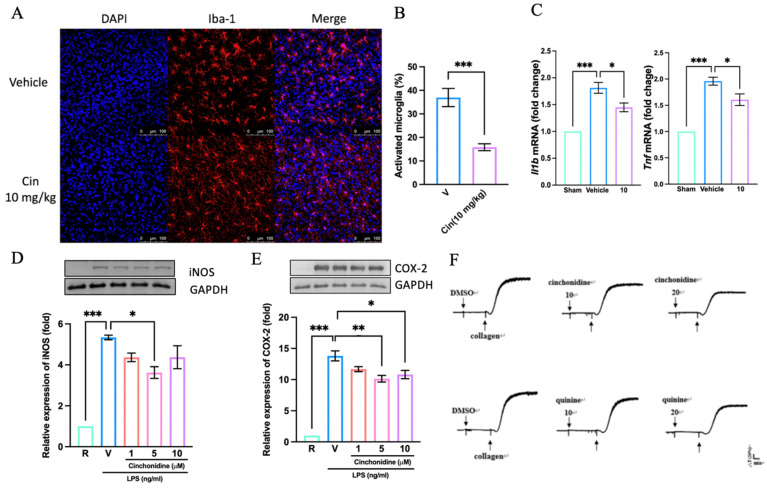
Cinchonidine suppresses neuroinflammatory responses without affecting platelet aggregation. The anti-inflammatory effects of cinchonidine were evaluated in vivo and in vitro. (**A**) Representative immunofluorescence images of Iba1-positive microglia (red) in the ipsilateral cortex of MCAO mice treated with vehicle (V), or cinchonidine (10 mg/kg). Nuclei were counterstained with DAPI (blue). Scale bar = 100μm. (**B**) Quantification of activated microglia based on Iba1 immunoreactivity within the ipsilateral cortex. Quantitative analysis was performed using ImageJ software and normalized to the total analyzed area. (**C**) Relative mRNA expression levels of the pro-inflammatory cytokines IL1-β and TNF-α in ischemic brain tissue quantified by quantitative real-time PCR (qPCR). (**D**–**E**) BV2 microglial cells were stimulated with 150 ng/mL lipopolysaccharide (LPS) in the presence or absence of cinchonidine. Protein expression levels of inducible nitric oxide synthase (iNOS) (**D**) and cyclooxygenase-2 (COX-2) (**E**) were determined by Western blotting and normalized to GAPDH. (**F**) Representative traces of collagen-induced platelet aggregation in washed human platelets pretreated with vehicle (DMSO), cinchonidine (10 or 20 μM), or quinine (10 or 20 μM). Cinchonidine significantly attenuated neuroinflammatory responses while exerting minimal effects on platelet aggregation. Data are presented as mean ± SEM. * *p* < 0.05, ** *p* < 0.01, *** *p* < 0.001 as indicated.

To determine whether cinchonidine modulates neuroinflammation following ischemic injury, microglial activation was assessed by Iba1 immunofluorescence staining. As shown in Figure 3A, MCAO induced pronounced microglial activation in the ipsilateral brain, whereas treatment with cinchonidine (10 mg/kg) markedly reduced the number of Iba1-positive activated microglia. Quantitative analysis confirmed a significant reduction in microglial activation in cinchonidine-treated mice compared with vehicle-treated MCAO mice (Figure 3B). To further investigate the anti-inflammatory effects of cinchonidine in vivo, the mRNA expression levels of the pro-inflammatory cytokines IL1-β and TNF-α (gene: *Il1b* and *Tnf)* were quantified by quantitative real-time PCR (qPCR). MCAO significantly increased the expression of both cytokines compared with sham controls, whereas cinchonidine treatment significantly attenuated these increases (Figure 3C), indicating suppression of the post-ischemic inflammatory response. The anti-inflammatory effects of cinchonidine were further examined in LPS-stimulated BV2 microglial cells. As shown in Figure 3D,E, LPS stimulation markedly increased the expression of inducible nitric oxide synthase (iNOS) and cyclooxygenase-2 (COX-2). Treatment with cinchonidine significantly reduced the expression of both inflammatory mediators, further supporting its anti-inflammatory activity in vitro. To evaluate whether cinchonidine influences platelet function, collagen-induced platelet aggregation was examined using washed human platelets. As shown in Figure 3F, cinchonidine (10–20 μM) did not significantly affect platelet aggregation compared with vehicle-treated controls. cinchonidine treatment did not significantly affect collagen-induced platelet aggregation compared with the vehicle control. Similarly, quinine treatment showed no obvious inhibitory effect under the tested conditions. These results suggest that cinchonidine does not exhibit significant antiplatelet activity.

### 3.4. Preservation of Blood–Brain Barrier Integrity and Attenuation of Apoptosis

**Figure 4:**
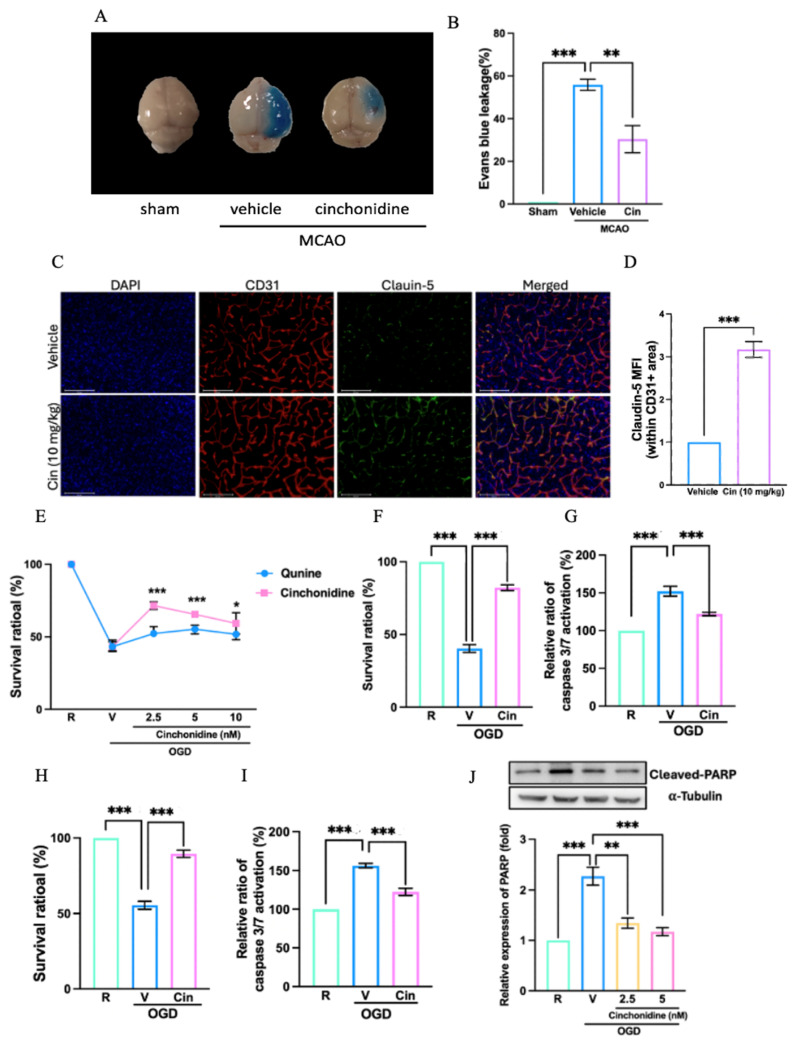
Preservation of blood–brain barrier integrity and attenuation of apoptosis. (**A**) Representative images of Evans blue extravasation in sham-operated mice and MCAO mice treated with vehicle or cinchonidine (10 mg/kg). (**B**) Quantification of Evans blue leakage as an indicator of blood–brain barrier permeability. (**C**) Representative immunofluorescence images of CD31-positive cerebral vessels (red) and the tight junction protein Claudin-5 (green) in the peri-infarct cortex. Nuclei were counterstained with DAPI (blue). Scale bar = 125μm. (**D**) Quantification of Claudin-5 mean fluorescence intensity (MFI) within CD31-positive vascular areas. (**E**) Survival of cerebral endothelial cells (CECs) subjected to oxygen–glucose deprivation (OGD) and treated with quinine or cinchonidine. (**F**) Cell viability of OGD-exposed CECs following cinchonidine treatment. (**G**) Caspase-3/7 activity in OGD-exposed CECs. (**H**) Cell viability of OGD-exposed Neuro2A neuronal cells. (**I**) Caspase-3/7 activity in OGD-exposed Neuro2A cells. (**J**) Western blot analysis and quantification of cleaved PARP expression in OGD-treated CECs, normalized to α-tubulin. Data are presented as mean ± SEM from at least three independent experiments. * *p* < 0.05, ** *p* < 0.01, *** *p* < 0.001 as indicated.

To determine whether cinchonidine protects blood–brain barrier (BBB) integrity following ischemic stroke, Evans blue extravasation was evaluated in MCAO mice. As shown in Figure 4A, MCAO induced substantial Evans blue leakage into the ipsilateral hemisphere, whereas cinchonidine treatment (10 mg/kg) markedly reduced dye extravasation. Quantitative analysis confirmed a significant reduction in BBB permeability in cinchonidine-treated mice compared with vehicle-treated MCAO mice (Figure 4B). To further assess endothelial integrity, the expression of the tight junction protein Claudin-5 was examined by immunofluorescence staining. MCAO markedly reduced Claudin-5 expression in CD31-positive cerebral vessels, whereas cinchonidine treatment preserved Claudin-5 immunoreactivity (Figure 4C). Quantitative analysis demonstrated a significant increase in Claudin-5 expression within the CD31-positive vascular area following cinchonidine treatment (Figure 4D), indicating preservation of BBB structural integrity. To investigate the direct cytoprotective effects of cinchonidine under ischemic conditions, cerebral endothelial cells (CECs) and Neuro2A neuronal cells were subjected to oxygen–glucose deprivation (OGD). In CECs, cinchonidine significantly improved cell survival compared with vehicle-treated OGD controls and exhibited greater protective effects than quinine at equivalent concentrations (Figure 4E). Consistent with these findings, cinchonidine significantly increased cell viability and reduced caspase-3/7 activation in OGD-exposed CECs (Figure 4F,G). Similar protective effects were observed in Neuro2A neuronal cells. Cinchonidine treatment significantly enhanced cell survival and attenuated caspase-3/7 activation following OGD exposure (Figure 4H,I). Furthermore, Western blot analysis demonstrated that cinchonidine reduced the expression of cleaved PARP, a marker of apoptosis, in OGD-treated CECs (Figure 4J). Collectively, these findings suggest that cinchonidine preserves BBB integrity and protects both endothelial and neuronal cells from ischemia-induced injury and apoptosis.

### 3.5. Cinchonidine Modulates Apoptosis-Related Markers in Ischemic Conditions

**Figure 5:**
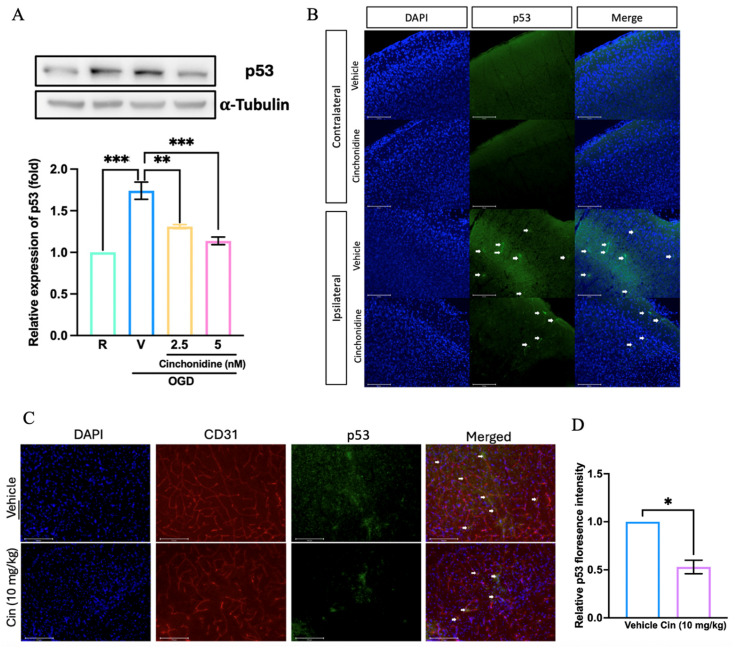
Cinchonidine Modulates Apoptosis-Related Markers in Ischemic Conditions (**A**) Protein expression of p53 in cerebral endothelial cells (CECs) subjected to oxygen–glucose deprivation (OGD) and treated with cinchonidine (2.5 or 5 nM), determined by Western blotting and normalized to α-tubulin. (**B**) Representative immunofluorescence images of p53 expression (green) in the contralateral and ipsilateral cortex following MCAO. Arrows indicate p53-positive cells within the ischemic region. Nuclei were counterstained with DAPI (blue). Scale bar: 125 μm (**C**) Representative double immunofluorescence staining of the endothelial marker CD31 (red) and p53 (green) in the peri-infarct cortex of vehicle- and cinchonidine-treated mice. Nuclei were counterstained with DAPI (blue). Scale bar: 125 μm (**D**) Quantification of p53 fluorescence intensity within CD31-positive vascular regions. Data are presented as mean ± SEM. * *p* < 0.05, ** *p* < 0.01, *** *p* < 0.001 as indicated.

To further investigate the molecular mechanisms underlying cinchonidine-mediated protection, p53-associated apoptotic signaling was examined. As shown in Figure 5A, OGD markedly increased p53 expression in cerebral endothelial cells, whereas cinchonidine treatment significantly reduced p53 protein levels in a dose-dependent manner. Consistent with these findings, immunofluorescence analysis demonstrated increased p53 immunoreactivity in the ipsilateral cortex following MCAO, which was attenuated by cinchonidine treatment (Figure 5B). Double immunofluorescence staining further revealed p53 expression within CD31-positive vascular structures, and cinchonidine significantly reduced p53 fluorescence intensity in the cerebral vasculature2 (Figure 5C,D). These findings suggest that cinchonidine mitigates ischemia-induced activation of p53-associated stress signaling, particularly within the cerebral endothelium.

## 4. Discussion

Despite previously reported anti-inflammatory and cytoprotective effects of cinchonidine, its role in ischemic stroke remains poorly understood. Its effects on endothelial function, BBB integrity, and neurovascular interactions have not been comprehensively investigated. Furthermore, it remains unclear whether cinchonidine can provide neuroprotection without affecting platelet aggregation, which is an important consideration given the risk of hemorrhagic complications associated with many stroke therapies. In the present study, we demonstrated that cinchonidine exerts robust protective effects against ischemic brain injury in both in vivo and in vitro models. Cinchonidine treatment significantly reduced infarct volume and brain edema while improving neurological outcomes following MCAO. These findings suggest that cinchonidine is associated with reduced ischemic brain injury and improved functional recovery following cerebral ischemia. Neuroinflammation is widely recognized as a critical contributor to secondary injury following ischemic stroke [24]. Activation of microglia and subsequent release of pro-inflammatory mediators, such as inducible nitric oxide synthase (iNOS) and cyclooxygenase-2 (COX-2), exacerbate neuronal damage and amplify tissue injury [17,25,26]. In this study, cinchonidine markedly suppressed microglial activation in vivo and reduced the expression of iNOS and COX-2 in LPS-stimulated BV2 cells. These findings are consistent with previous reports describing the anti-inflammatory properties of cinchonidine and related cinchona alkaloids. In addition, cinchonidine significantly reduced the mRNA expression levels of the pro-inflammatory cytokines TNF-α and IL-1β in ischemic brain tissue. However, because reduced infarct severity may itself secondarily attenuate inflammatory responses, our findings should be interpreted as demonstrating an association between cinchonidine treatment and reduced neuroinflammation rather than establishing inflammation as the primary therapeutic mechanism. Among all findings presented in this study, preservation of endothelial integrity and blood–brain barrier (BBB) function emerged as the most consistent feature associated with cinchonidine-mediated neuroprotection. The BBB is essential for maintaining central nervous system homeostasis, and its disruption is a hallmark of ischemic stroke. Increased BBB permeability leads to vasogenic edema, infiltration of peripheral immune cells, and further neuronal injury [27]. In the present study, cinchonidine significantly reduced Evans blue extravasation, indicating preservation of BBB integrity. Moreover, cinchonidine preserved Claudin-5 expression within CD31-positive cerebral vessels, further supporting maintenance of BBB structural integrity after ischemic injury. In parallel, cinchonidine enhanced cell viability and reduced apoptosis in primary cerebral endothelial cells under oxygen–glucose deprivation (OGD) conditions. Together, these findings support a strong association between endothelial protection, BBB preservation, and the neuroprotective effects observed following cinchonidine treatment. Together, these findings support a strong association between endothelial protection, BBB preservation, and the neuroprotective effects observed following cinchonidine treatment.

Endothelial cells are essential components of the neurovascular unit (NVU), which also includes neurons, astrocytes, pericytes, and extracellular matrix components that collectively maintain cerebral homeostasis [28,29,30]. Disruption of the NVU during ischemia leads to BBB breakdown, neuroinflammation, and neuronal death [31]. Among these components, endothelial cells play a particularly critical role by regulating vascular permeability, leukocyte adhesion, and inflammatory signaling [32]. Therefore, targeting endothelial dysfunction may provide a more comprehensive therapeutic strategy compared with approaches that focus solely on neurons [33]. The ability of cinchonidine to preserve endothelial function suggests that it may act at the level of the NVU, thereby providing broader neurovascular protection.

From a mechanistic standpoint, our results demonstrate that cinchonidine attenuates p53-associated stress signaling in both endothelial cells and ischemic brain tissue. Furthermore, p53 immunoreactivity was detected within CD31-positive vascular structures, suggesting that endothelial stress responses may contribute to ischemia-induced vascular injury. While p53 is traditionally viewed as a key regulator of cellular stress responses, its downregulation here serves as a significant indicator of reduced apoptotic signaling. These findings align with previous observations of cinchonidine’s cytoprotective effects but extend our understanding into the context of neurovascular protection. Rather than acting on a single target, cinchonidine likely exerts its effects through the broader stabilization of the endothelial cell environment, as evidenced by the preservation of BBB integrity. A critical challenge in current stroke therapy is the delicate balance between efficacy and hemostatic safety. Standard interventions like rt-PA and antiplatelet agents, while effective in recanalization, carry a substantial risk of hemorrhagic transformation [34,35]. In this regard, our finding that cinchonidine provides robust neurovascular protection without significantly interfering with platelet aggregation is of particular interest. This observation suggests that the observed neurovascular benefits are independent of systemic antiplatelet activity. Unlike conventional antithrombotic agents that carry an inherent risk of intracranial hemorrhage, cinchonidine-associated neurovascular protection appears to be closely linked to preservation of BBB integrity, together with modulation of inflammatory and apoptotic responses.

**Figure 6:**
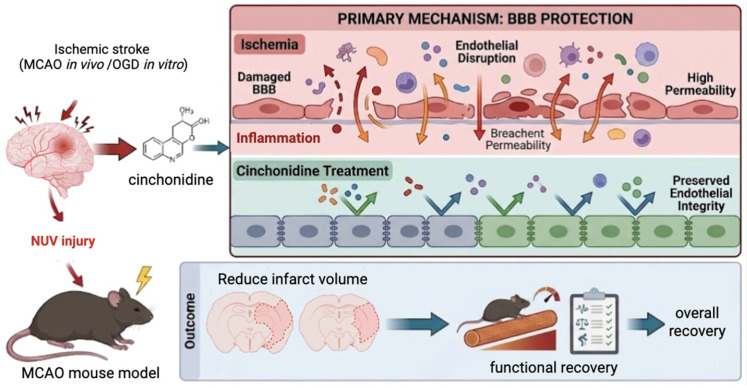
Proposed neurovascular protective mechanism of cinchonidine. Schematic depicts the integrated therapeutic actions of cinchonidine within the ischemic neurovascular unit. Following an ischemic insult, the activation of microglia and the induction of apoptosis in cerebral endothelial cells lead to severe neuroinflammation and blood–brain barrier (BBB) disruption. Cinchonidine counteracts these pathological processes by suppressing microglial activation and preserving endothelial cell survival. These protective actions effectively maintain BBB integrity and reduce brain edema without interfering with platelet aggregation, thereby exhibiting potent neurovascular protection with a favorable hemostatic safety profile.

In summary, our findings provide a comprehensive model of cinchonidine’s multi-target actions, as illustrated in Figure 6. By preserving BBB integrity and endothelial function while simultaneously attenuating inflammatory and apoptotic responses, cinchonidine serves as a promising therapeutic lead that provides robust neurovascular protection with a favorable hemostatic safety profile. From a drug discovery perspective, the pleiotropic properties of cinchonidine—encompassing anti-inflammatory, anti-apoptotic, and endothelial-protective actions—offer a comprehensive advantage over single-target agents. As a naturally derived quinoline alkaloid, cinchonidine represents a structurally diverse lead compound with an established safety window in other contexts. Its ability to concurrently protect the neurovascular unit and suppress microglial activation (as seen by reduced iNOS and COX-2) highlights its potential as a multi-target therapeutic candidate that addresses the complex pathophysiology of ischemia–reperfusion injury. Finally, several limitations of this study warrant mention. First, although a post-treatment group was included, the majority of experiments were conducted using a pre-treatment paradigm, which may not fully reflect clinical treatment scenarios in acute ischemic stroke. Second, cerebral blood flow was not continuously monitored during MCAO procedures, and therefore inter-animal variability in ischemic severity cannot be completely excluded. Third, only male mice were included in this study, and potential sex-dependent differences in the neuroprotective effects of cinchonidine remain unknown. Fourth, while our results demonstrate significant protection during the acute phase of ischemic injury, the follow-up period was relatively short, and longer-term functional recovery and tissue remodeling were not evaluated. Fifth, the pharmacokinetic properties, blood–brain barrier penetration, metabolic stability, and tissue distribution of cinchonidine were not investigated While platelet aggregation assays suggested that cinchonidine does not significantly interfere with hemostatic function under the experimental conditions tested, a more comprehensive evaluation of coagulation parameters and bleeding risk is warranted. Furthermore, dedicated toxicity studies examining the effects of the vehicle formulation and prolonged exposure to cinchonidine on cellular and platelet function were not performed and should be investigated in future studies. Finally, while p53-associated signaling was examined in the present study, the precise upstream regulators and additional apoptotic mediators, such as Bcl-2, Bax, and caspase-9, warrant further investigation. Future studies should also explore the potential interactions between cinchonidine and established reperfusion therapies, including rt-PA, to better define its translational potential in ischemic stroke. In addition, representative morphological analyses of OGD-treated cerebral endothelial cells and neuronal cells, as well as live/dead staining assays, may provide further insight into the cellular protective effects of cinchonidine and should be explored in future studies.

## Figures and Tables

**Table 1:** Primer sequences used for RT-qPCR.

Primer	Forward (Sense)	Reverse (Antisense)
IL-1β	CTCATTGTGGCTGTGGAGAA	CACACACCAGCAGGTTATCA
TNF-α	CATCTTCTCAAAACTCGAGTGACAA	TGGGAGTAGATAAGGTACAGCCC

## Data Availability

All data generated or analyzed during this study are included in this published article.
